# Pho-Tip: One-Pot Dephosphorylation for Rapid and Sensitive Analysis of DIA Phosphoproteomics Data

**DOI:** 10.1021/acs.analchem.5c07139

**Published:** 2026-02-23

**Authors:** Katharina D. Faisst, Kate Lau, Ludwig R. Sinn, Lukasz Szyrwiel, Vadim Demichev

**Affiliations:** † Quantitative Proteomics Laboratory, Department of Biochemistry, 14903 Charité − Universitätsmedizin Berlin, Berlin 10117, Germany; ‡ Biochemistry and Systems Biology of the Metabolism Laboratory, Department of Biochemistry, Charité − Universitätsmedizin Berlin, Berlin 10117, Germany

## Abstract

Recent advances in instrumentation and data processing have transformed data-independent acquisition (DIA) proteomics into a reliable technology for quantitative profiling of post-translational modifications. However, analysis of DIA phosphoproteomics data is challenging due to the large search space, wherein all combinations of phosphosites on a peptide need to be considered. Current approaches therefore face significant hurdles in detecting low-abundant phosphorylated peptides, in particular when working with low sample amounts. Here we introduce Pho-Tip, a lossless one-pot dephosphorylation strategy. We show that Pho-Tip enables comprehensive mapping of phosphorylated peptide sequences, facilitating streamlined creation of experiment-focused *in silico* predicted spectral libraries and thus rapid and sensitive analysis of DIA phosphoproteomics experiments.

## Graphical abstract

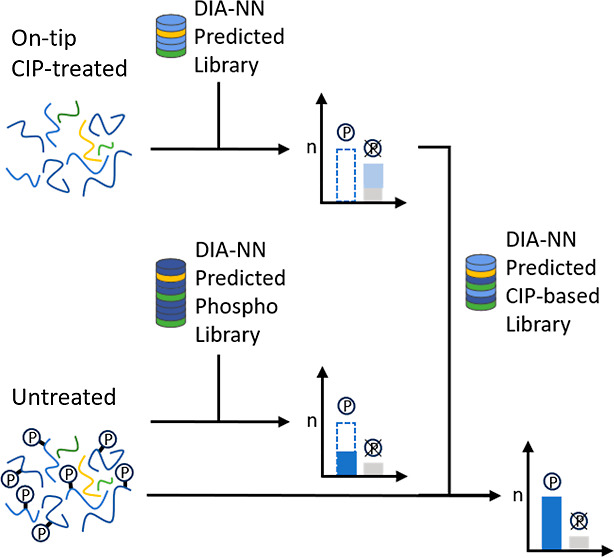

## Introduction

Data-independent acquisition (DIA) proteomics has gained significant popularity in recent years, promoted by step change improvements in mass spectrometry instrumentation and data analysis software algorithms.^1,2^ DIA has also been established for the analysis of post-translational modifications (PTMs), including phosphorylation,^3^ with comprehensive algorithms also developed to enable confident site localization.^4,5^ At the same time, the accepted gold standard approach to phosphoproteomics involves spectral library creation via deep offline fractionation-based analysis of a pooled sample.^6,7^ Given that this process is laborious and is not guaranteed to enable full coverage of detectable phosphosites throughout the experiment, library-free analysis of DIA phosphoproteomics data has emerged as a viable alternative, enabled by the advances in DIA data processing software.^8,9^

This approach, however, results in a large search space, as a single peptide sequence can give rise to multiple possible combinations of occupied phosphosites. The large search space reduces sensitivity, since the DIA software necessarily has to impose stricter quality requirements on peptide spectrum matches in order to report them as confidently identified. Further, the analysis time grows along with the search space, presenting a considerable computational burden, this being particularly relevant in view of the increasing proportion of large-scale proteomics experiments among the applications of DIA.^10,11^

To address the search space problem of DIA phosphoproteomics, we introduce Pho-Tip, a one-pot dephosphorylation strategy that leverages phosphopeptide enrichment followed by alkaline phosphatase treatment on EvoTips (Evosep) and liquid chromatography coupled to mass spectrometry (LC–MS) analysis. Subsequent search against the full sequence database provides comprehensive identification of any amino acid sequences which may bear a phosphorylation. These sequences can then serve as the basis for predicting compact phosphopeptide-containing spectral libraries for the sensitive analysis of phosphoproteomics experiments. Here, we demonstrate that this strategy outperforms the conventional search against the full sequence database in terms of speed and coverage.

## Methods

### Sample Preparation - HeLa

HeLa cells were cultured in Dulbecco’s Modified Eagle’s Medium (DMEM) medium (500 mL) containing l-glutamine, supplemented with 10% fetal calf serum (FCS) and 1% Penicillin–Streptomycin. Cells were maintained at 37 °C in a humidified atmosphere with 5% CO_2_. The cultures were washed with PBS and harvested using ethylenediaminetetraacetic acid (EDTA). Cells were then washed with PBS containing 0.8 mM PMSF, aliquoted, pelleted (5 × 10̂6 cells per pellet) and stored at −80 °C. Cells were thawed, washed with ice-cold Milli-Q water and afterward resuspended in lysis buffer (0.1 M TRIS-HCl, 8 M Urea) containing PhosphataseArrest I. The sample was vortexed and sonicated in an ultrasonic bath for 30 s. Homogenization was performed using a 26G needle. Subsequently, benzonase was added, and the mixture was incubated at RT for 15 min. After incubation, the mixture was centrifuged at 14,800 rpm for 60 min, and the supernatant was collected. Protein concentration was then determined using the Pierce 660 nm Protein Assay (Thermo Fisher Scientific, Waltham, MA, USA).

### Sample Preparation - Yeast

Yeast strain BY4741ki^12^ was cultured in Yeast Extract Peptone Dextrose (YPD) medium at 30 °C, 270 rpm. The cultures were harvested by centrifugation with 3,000*g* for 15 min at 4 °C. Pellets were washed with 1× phosphate buffered saline (PBS) containing 0.8 mM phenylmethylsulfonyl fluoride (PMSF), aliquoted and stored at −80 °C. Cells were thawed, washed with ice-cold Milli-Q water and pretreated with 0.2 M sodium hydroxide (NaOH) for 10 min on ice. After pelleting, cells were resuspended in lysis buffer (7 M Urea, 0.1 M ammonium bicarbonate (ABC)) containing PhosphataseArrest I (G-Biosciences, St. Louis, MO, USA), mixed with 0.5 mm glass beads, and subjected to lysis by bead-beating using the SPEX CertiPrep Geno/Grinder 2 (SPEX SamplePrep, Metuchen, NJ, USA) at 1500 rpm for four 5 min cycles, with cooling on ice between cycles. The samples were centrifuged at 20,000*g* for 15 min at 4 °C, and the supernatants were collected, and centrifuged again at 20,000*g* for 1 h at 4 °C. The final supernatant was used for protein concentration determination using the Pierce BCA Protein Assay Kit (Thermo Fisher Scientific, Waltham, MA, USA).

For disulfide reduction, the sample was incubated with 5 mM dithiothreitol (DTT) for yeast (10 mM for HeLa) at RT for 30 min, then kept on ice for 5 min. Alkylation was performed by adding 10 mM iodoacetamide (IAA) for yeast (20 mM for HeLa), followed by incubation in the dark for 20 min. The reaction was quenched with an additional 5 mM DTT for yeast (10 mM for HeLa), and incubated in the dark for 10 min. The cell lysate was diluted 1:5 with 0.1 M ABC, and trypsin was added at a 1:100 enzyme-to-protein ratio. The mixture was vortexed, centrifuged, and incubated overnight at 37 °C. Digestion was stopped by acidifying with trifluoroacetic acid (TFA) to pH 2–3. After 15 min incubation at RT, the samples were centrifuged at 4 °C, and the supernatants were collected.

The yeast and HeLa samples were desalted using STAGE-Tips according to the protocol.^13^ STAGE-Tips were activated with methanol (MeOH), and washed with 60% (v/v) acetonitrile (ACN) and 0.1% (v/v) TFA. Samples were loaded onto the tips, and washed with 0.1% TFA. Peptides were eluted with 60% ACN. The eluates were dried using the Eppendorf Concentrator Plus (Eppendorf SE, Hamburg, Germany) at 45 °C, and reconstituted in 2% ACN, 0.1% TFA. Peptide concentrations were determined using the Implen NanoPhotometer N60/N50 (Implen GmbH, München, Germany). Samples were stored at −80 °C.

### Phosphopeptide Enrichment Using the High-Select TiO_2_ Phosphopeptide Enrichment Kit and Phosphatase Reaction - HeLa

Phosphopeptide enrichment was performed with the High-Select TiO_2_ Phosphopeptide Enrichment Kit (Thermo Fisher Scientific, Waltham, MA, USA) according to the manufacturer’s instructions. In brief, dried peptide pellets (3 replicates per condition) were resuspended in binding/equilibration buffer. Columns were first washed before equilibration, both with centrifugation at 3000*g* for 2 min. The sample was applied and loaded at 1000*g* for 5 min. The flow-through was reapplied to ensure full binding, with the same centrifugation and retention of the flowthrough. Afterward, the column was washed by adding first Binding/Equilibration and then wash buffer, followed by centrifugation at 3000*g* for 2 min. These two washing steps were repeated, and the column was then washed with LC–MS grade water. To elute the phosphopeptides, 25 μL elution buffer was added. The column was centrifuged at 1000*g* for 5 min, and the elution step was repeated with 25 μL elution buffer. The eluates were dried using the Eppendorf Concentrator Plus at 45 °C, and reconstituted in 2% ACN, 0.1% TFA. Peptide concentrations were determined using Nanodrop.

For phosphatase reaction the peptide lysate was first mixed with 1× calf intestinal alkaline phosphatase (CIP) buffer (Promega, Madison, WI, USA), then divided equally between two tubes for each replicate. For CIP (Promega, Madison, WI, USA)-treated samples, CIP was added to one of the tubes while the other tube without CIP was considered a mock sample. The reaction was carried out by incubating both mock- and CIP-treated samples at 37 °C for 2 h. Following incubation, samples were quenched by adding TFA to a final concentration of 0.5%.

The samples were again desalted using STAGE-Tips according to the protocol. STAGE-Tips were activated with MeOH, and washed with 60% ACN and 0.1% TFA. Samples were loaded onto the tips, and washed afterward with 0.1% TFA. Peptides were eluted with 60% ACN. The eluates were dried using the Eppendorf Concentrator Plus at 45 °C, and reconstituted in 2% ACN, 0.1% TFA. Peptide concentrations were determined using Nanodrop. Samples were stored at −80 °C.

### Phosphopeptide Enrichment with MagReSyn Zr-IMAC HP Beads and Phosphatase Reaction - Yeast

Phosphopeptide enrichment of yeast proteome digests was performed using MagReSyn Zr-IMAC HP beads according to the manufacturer’s instructions. Dried peptide pellets were resuspended in binding buffer. MagReSyn Zr-IMAC HP beads were equilibrated by resuspending in 500 μL binding buffer for three cycles. The equilibrated beads (5 μL) were distributed into wells and 30 μg of resuspended peptides were added (6 replicates per condition). The mixture was incubated mixing on an Eppendorf ThermoMixer C (Eppendorf SE, Hamburg, Germany) at 750 rpm for 20 min. Beads were washed sequentially with binding buffer, Wash Solvent 1 (80% ACN +1% TFA), and Wash Solvent 2 (10% ACN +0.2% TFA). Phosphopeptides were eluted from the beads using 50 μL of 0.5%, 1%, and 2% NH_4_OH solutions, pooled, and the pH was adjusted to 9–10 with 10% TFA. For the following dephosphorylation reaction, 10× CIP buffer was added to achieve a final concentration of 1×.

EvoTips were prepared by activation and equilibration according to the manufacturer’s protocol. Peptide samples (30 ng) were loaded per EvoTip. For mock samples, the loaded EvoTips were spun down immediately without further treatment. For CIP-treated samples, CIP was added to the loaded EvoTips, followed by incubation at 37 °C for 2 h. After incubation, CIP-treated samples were quenched with 20% formic acid (FA). All EvoTips were subsequently washed with 0.1% FA and then loaded with 100 μL of 0.1% FA in preparation for analysis.

### LC–MS/MS Analysis - HeLa

Samples were acquired in data-independent acquisition (DIA)/Zeno-SWATH mode, on a ZenoTOF 7600+ mass spectrometer (SCIEX, Toronto, Canada) coupled online to an Acquity M-Class UPLC system (Waters, Milford, MA, USA). In each measurement we injected 200 ng of sample onto a reversed-phase chromatography nanoEase M/Z HSS T3 column (100 Å, 1.8 μm, 0.3 × 150 mm) with buffers A (0.1% FA) and B (acetonitrile with 0.1% FA) ramping from 3 to 40% buffer B at a flow rate of 5 μL/min and at 35 °C using a 30 min total gradient as described in Wang *et al.* 2022.^14^

The mass spectrometric method was similar to the one employed by Wang and colleagues^14^ with the following exceptions: 85 variable isolation windows between 4 and 8 Th were defined. The spray voltage was set to 5000 V and a scheduled ionization between minutes 2.5 and 22 was used.

### LC–MS/MS Analysis - Yeast

LC–MS was performed using an Evosep One system coupled to a TimsTOF ULTRA mass spectrometer equipped with a Captive Spray II ion source. Peptide separation was conducted on an 8 cm × 150 μm ID Performance Column with 1.5 μm particle size, maintained at 40 °C. A standard 60 SPD Evosep method was applied, utilizing a 21 min gradient for a total sample-to-sample time of 24 min. Solvent A consisted of 0.1% FA, and Solvent B was ACN with 0.1% FA (Optima LCMS grade, Thermo Fisher Scientific, Waltham, MA, USA).

Data were acquired using a dia-PASEF scheme, optimized for a cycle time of 1.18 s. The isolation window scheme spanned *m*/*z* 388–1168 and 1/K0 0.67–1.28, covering most charge 2 precursor ions, using 28 × 27.8 Th windows. The acquisition utilized 100 ms accumulation and ramp times. The mass spectrometer was operated in “high sensitivity detection” mode, optimized for low sample amounts. The utilized acquisition scheme and the *m*/*z* range correspond to commonly used dia-PASEF settings. Specifically in case of phosphoproteomics, available data indicates that the precursor *m*/*z* range from 1168 to 1400 hosts only a small proportion of detectable phosphorylated precursor ions^7^ (7.4%), while extending the *m*/*z* range typically has detrimental overall effects on a timsTOF instrument's performance due to the associated decrease in the resolution in the IM space. Thus, the upper *m*/*z* limit in the 1000 to 1200 *m*/*z* range remains a popular choice for dia-PASEF. The dia-PASEF window scheme was selected to balance sensitivity and coverage.

### *In Silico* Predicted Spectral Library Generation

To generate full-proteome *in silico* predicted spectral libraries in DIA-NN 2.0.2, default settings were used, except the number of missed cleavages was set to 2, the precursor charge range was set to 2 to 3, the precursors’ *m*/*z* range was set to 400 to 1200. For libraries that include phosphorylation on Serine, Threonine, and Tyrosine as a variable modification (all analyses except CIP-based spectral library generation, as described in the next section), the maximum number of allowed variable modifications per peptide was set to 3.

### CIP-Based Spectral Library Generation

CIP-treated samples were analyzed in DIA-NN 2.0.2 using an *in silico* predicted library. In this case no variable modifications were specified. Scoring was set to Generic, and the FDR cutoff was set to 5%, to maximize coverage. The resulting empirical library in the .skyline.speclib format was then used as input to construct the predicted phospho library. For this step, the CIP-based library was added to DIA-NN in place of a FASTA database using the “Add FASTA” option, while--cut was provided in the Additional options field to disable enzymatic digestion of library peptide sequences. Phosphorylation was enabled as a variable modification, with up to three phosphorylations allowed per peptide. This resulted in a library that contains all possible phosphorylation states of peptide sequences that were detected in CIP-treated samples. This method thus generates a tailored spectral library, focusing on phosphorylated peptides from the data set, instead of relying on a generic protein database.

### Raw Data Analysis - HeLa and Yeast

DIA-NN 2.0.2 was used to analyze the experiment, the full settings are reflected by the logs deposited to the PRIDE repository. Briefly, default settings were used with MBR enabled, except the mass accuracy was set to 15 ppm (timsTOF) or 20 ppm (ZenoTOF); MS1 accuracy was 15 ppm (timsTOF) or 12 ppm (ZenoTOF). Scan window was inferred automatically for yeast data or set to 7 for human data. Calibration mass accuracy was set to 25 ppm for human data. Quantification mode was set to Legacy. A default 1% FDR threshold was applied for all steps except the generation of CIP-based library as indicated in the above section. Likewise, all steps relied on predicted libraries with phosphorylation specified as variable modification, except for CIP-based library generation.

## Results and Discussion

In the past, dephosphorylation has already been considered for the purpose of enhancing phosphopeptide detection, in combination with data-dependent acquisition (DDA) mass spectrometry. However, the inherent stochastic nature of DDA tends to result in a high missing value rate, and, consequently, early experiments were not able to achieve comprehensive phosphoproteome coverage.^15,16^ We speculated that high-sensitivity DIA proteomics, in contrast, can comprehensively capture the phosphoproteome via the analysis of a dephosphorylated sample.

To confirm this, we have enriched phosphopeptides from a HeLa cell line tryptic proteome digest using TiO_2_ columns (Thermo Fisher Scientific), followed by treatment with calf intestinal alkaline phosphatase (CIP) or left untreated (mock). The samples were then analyzed on a ZenoTOF 7600+ mass spectrometer (SCIEX), using a 20 min active chromatographic gradient (Methods). First, the data were searched with DIA-NN 2.0.2 against the human sequence database, with up to three phosphorylations enabled, confirming that the CIP treatment successfully removes the vast majority of peptide phosphorylations (Figure S1a).

We next analyzed the nature of possibly “CIP-resistant” phosphopeptides, that is peptides that are still detected as phosphorylated in CIP-treated samples. Motif analysis revealed that these exhibited less acidic contexts and elevated tyrosine phosphorylation compared to the full phosphoproteome (Figure S2a,b), albeit without clear patterns distinguishing them. The CIP-resistant peptides also demonstrated high intensities in mock samples compared to peptides that were not detected as phosphorylated upon CIP treatment (Figure S2c). We also analyzed the MS1 signals attributed to CIP-resistant peptides, but could not observe a reduction of intensities upon CIP-treatment, possibly due to poor quantitative data quality for the very few peptides detected (Figure S2d).

We then searched the CIP-treated sample against a database devoid of any variable peptide modification, resulting in a compact search space size for maximum identification sensitivity. In support of our hypothesis, we observed that the CIP-treated sample allowed the detection of 94% of amino acid sequences identified as phosphorylated in the mock sample (*n* = 7119). Moreover, the total number of detected peptide sequences, regardless of the phosphorylation status, increased from 15,014 to 27,962 (Figure S3) upon CIP treatment, hinting at potentially higher sensitivity of peptide detection in their dephosphorylated form. To investigate the possible reasons for the increased sensitivity beyond the search space reduction, we compared the MS1 signal attributed to each amino acid sequence in its phosphorylated form in the mock sample to the respective MS1 signal detected in the CIP-treated sample. Jointly detected amino acid sequences devoid of any phosphosites served as loading control. This analysis revealed a minor increase in detected signal following dephosphorylation, likely reflecting better average ionization efficiency of dephosphorylated peptides (Figure S4).

Next, we established Pho-Tip, a streamlined pipeline that enables to easily obtain the set of phosphopeptide amino acid sequences for the vast majority of phosphoproteomics applications (Figure 1a). For this, we coupled enrichment using MagReSyn Zr-IMAC HP magnetic beads (ReSyn Biosciences) to peptide dephosphorylation directly on EvoTips (Evosep). While we used TiO_2_ column-based enrichment for the proof-of-concept HeLa experiment, here we switched to a bead based approach, given that it is suitable for a wide range of peptide loads,^17^ down to 2.5 μg of peptide input in case of Zr-IMAC HP,^18^ expanding the applicability of our workflow. Recent work demonstrated how EvoTips can be used for lossless sample preparation via on-tip reactions,^19^ leading us to speculate that we can perform effective dephosphorylation on-tip, too. To evaluate the possible losses associated with it, we have carried out phosphopeptide enrichment based on 500 μg of yeast (*S. cerevisiae*) tryptic proteome digest and loaded the enriched phosphopeptides on EvoTips, subsequently either spinning them down immediately (i.e., mock) or first treating them with CIP for 2 h (Methods), prior to analysis on Evosep One (Evosep) coupled to a timsTOF Ultra (Bruker) mass spectrometer using a 60 SPD (samples per day) method (Methods). There was no significant difference in the detected intensities of peptides that do not contain serines, threonines or tyrosines, confirming that the CIP treatment on-tip can be considered lossless (Figure S5). As in the proof-of-concept experiment, CIP effectively dephosphorylated peptides (Figure S1b). Further, in the case of Pho-Tip, the analysis of MS1 signals attributed to CIP-resistant peptides confirmed almost complete dephosphorylation (median 7× reduction in intensity, Figure S2d). Analyzing the sequence coverage obtained with the CIP-treated sample, we again observed 94% of sequences detected as phosphorylated in the mock sample. Upon CIP treatment, detected peptide sequences rose from 14,711 to 27,637 (Figure S3).

**Figure 1:**
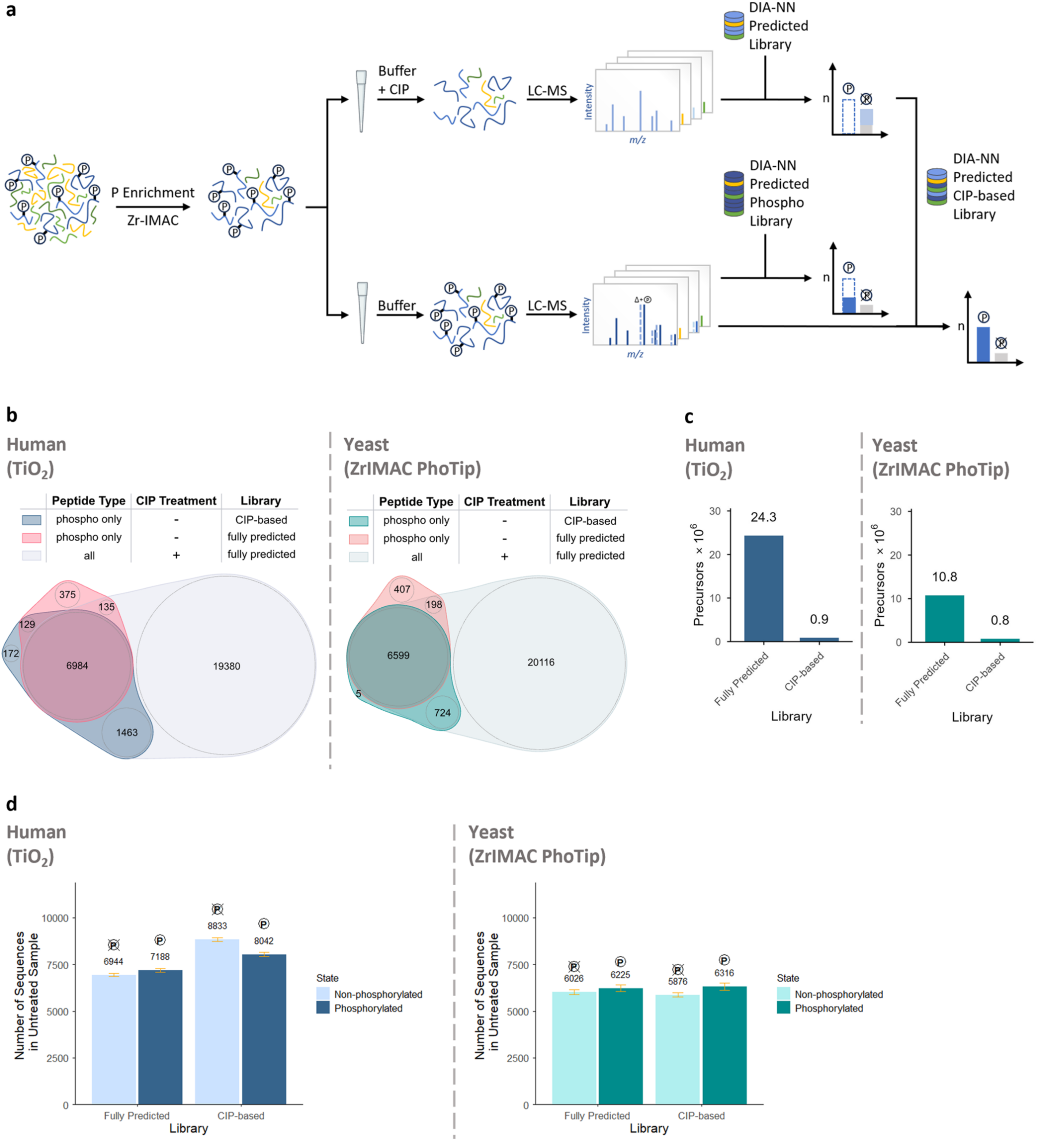
Pho-Tip improves phosphoproteomics sensitivity and boosts data analysis speed. (a) Pho-Tip experimental workflow including phosphopeptide enrichment and CIP-treatment on-tip, analysis on Evosep One coupled to timsTOF Ultra and searching of phosphopeptide samples exclusively against sequences found in CIP-treated samples, compared to searching against the full sequence database. (b) Overlap of phosphorylated peptide sequences identified in mock samples, analyzed with either fully predicted (red) or CIP-based library (blue/green), and peptide sequences in CIP-treated samples analyzed with fully predicted library (gray), separately for TiO_2_ as well as Zr-IMAC coupled to Pho-Tip. (c) Precursor counts for the full sequence database–based and CIP-based *in silico* predicted libraries, reflecting the analysis speed. (d) Number of identified sequences split by phosphorylation state (phosphorylated vs non-phosphorylated) comparing full sequence database–based and CIP-based library search, separately for TiO_2_ as well as Zr-IMAC coupled to Pho-Tip.

Of note, the coefficients of variation (CVs) observed for peptides in Pho-Tip or TiO_2_-enriched CIP-treated samples data appeared comparable to those observed without CIP treatment (Figure S6), supporting the robustness of the CIP treatment workflow.

We then proceeded to investigate how searching phosphopeptide data (that is, generated from samples without CIP treatment) against sequences exclusively identified in CIP-treated samples affects the numbers of detected phosphopeptides, compared to searching against the fully predicted sequence database of the respective species (Figure 1b). Here, setting the maximum number of variable modifications to 3, allowing 2 missed cleavages and precursor charges 2–3 resulted in spectral libraries of the size ∼896 k precursors (human) and ∼791 k precursors (yeast), compared to ∼24.3 M and ∼10.8 M precursors, respectively, in case of the full sequence search space (Figure 1c). Searching the phosphopeptide samples, we observed that CIP-based predicted libraries resulted in identification of about +15% on average for the human and about +2% for the Pho-Tip-processed yeast samples (Figure 1d). A possible reason for the smaller identification benefit observed for yeast could be the lower complexity of the yeast phosphoproteome and the more limited benefit of the CIP-based library restriction in this context. The gains appeared distributed across the whole range of precursor masses and retention times (Figure S7). To evaluate the ultimate sensitivity of the LC–MS setup and sample preparation workflow, we also examined the results with MBR disabled for the analysis of identified sequences comparing full sequence databas–based and CIP-based library searches (Figure S8), with a similar marginal advantage observed for the latter.

In this work, we tackle the computational challenge of phosphoproteomics: a large search space when considering all possible phosphopeptides that results in decreased sensitivity for phosphopeptide identification as well as a demand for large amounts of computational resources. While the creation of spectral libraries via offline fractionation of a pooled sample is an option,^6,7^ it may be too labor-intensive to perform it for each project, and it further requires significant sample amounts to be effective. In addition, peptides with rare phosphosite occupancy configurations may be missed using the pooled library approach.

We propose a novel, “biochemical”, solution: the search space is reduced in-sample, by enzymatically dephosphorylating all phosphopeptides, wherein multiple peptide species are converted into a single unmodified peptide sequence. Sensitivity for phosphopeptide sequence detection is maximized due to the fact that Pho-Tip is a lossless reaction and the detectability of unmodified peptides compared to phosphopeptides is improved. The identified peptide sequences then allow the creation of experiment-focused *in silico*-predicted spectral libraries that achieve fast and comprehensive analysis of regular, untreated phosphopeptide-enriched samples. We note that the use of standardized kits and reagents as well as standard MS instrumentation allows a wider accessibility to the proteomics community.

We note that the benefits of Pho-Tip may extend beyond just speeding up the analysis and increasing its sensitivity. In fact, in Pho-Tip each phosphopeptide sequence is detected twice, in modified and unmodified form. It is natural to assume that such repeated detection would result in a lower false discovery rate at the same *q*-value reported by the software, possibly allowing to filter the data at a *q*-value threshold less stringent than the commonly accepted 1%. We will leave it for future investigations to assess the magnitude of this effect with a suitable experiment design.

We envision that Pho-Tip can be used in a number of distinct ways as part of a phosphoproteomics workflow with the option for automation. For example, either a subset of samples or a pooled sample may be analyzed via Pho-Tip, to create the spectral library. The latter approach is conceptually similar to spectral library generation via offline fractionation but avoids the associated requirements for large sample amounts.

Alternatively, we speculate that Pho-Tip can serve not just as a spectral library creation method, but can also be used directly in quantitative proteomics experiments. While in many cases knowing the exact phosphosite location is essential in the context of the experiment, the numbers of sites confidently localized are typically low,^4,5^ often fold-change lower than the numbers of detected phosphopeptides. This leads to the 75% confidence threshold, as reported by the analysis software, often being used as the basis of phosphosite identification and subsequent quantification, but even with such a loose filtering many sites are being discarded. We note that it is common to perform preliminary discovery screens that are then followed by validation based on selected samples of interest, at a lower scale and possibly higher sample amounts and longer chromatographic gradients, possibly combined with sensitive parallel reaction monitoring (PRM), resulting in improved data quality. We speculate that in some cases discovery screens may not require resolution down to a specific site, but rather could focus on detection of differentially regulated peptide species, with subsequent investigation uncovering the exact sites involved. Such a design would benefit from maximum possible sensitivity, making Pho-Tip an ideal building block for a candidate screening method, complementary to the conventional site-resolved phosphoproteomics approaches.

## Conclusion

In this study, we addressed the search space problem in phosphoproteomics by introducing Pho-Tip, a one-pot dephosphorylation strategy that enables creation of experiment-specific spectral libraries. The new approach is straightforward to deploy in a proteomics lab and results in an order of magnitude smaller search spaces, promoting accessibility and wider adoption of phosphoproteomics.

## Supplementary Material







## Data Availability

The MS proteomics data have been deposited to the ProteomeXchange Consortium via the PRIDE^20^ partner repository with the data set identifier PXD070420.

## Notes

Number of phosphorylated and non-phosphorylated precursors to show efficient desphosphorylation, separately for TiO_2_ as well as Zr-IMAC coupled to Pho-Tip (Figure S1); analysis of motifs and peptide abundance of “CIP-resistant” phosphopeptides, separately for TiO_2_ as well as Zr-IMAC coupled to Pho-Tip (Figure S2); overlap of peptide sequences regardless of phosphorylation status in mock samples, analyzed with either fully predicted or CIP-based library, and peptide sequences in CIP-treated samples analyzed with fully predicted library, separately for TiO_2_ as well as Zr-IMAC coupled to Pho-Tip (Figure S3); density distribution of MS1 signal differences between unphosphorylated peptide sequences detected in CIP-treated samples and their phosphorylated counterparts in untreated samples for human TiO_2_-enriched samples (Figure S4); density distribution of MS1 signal differences between unphosphorylated peptide sequences detected in CIP-treated samples and their phosphorylated counterparts in untreated samples for Pho-Tip samples (Figure S5); precursor-level CV values for jointly detected precursors by treatment, separately for TiO_2_ as well as Zr-IMAC coupled to Pho-Tip (Figure S6); precursor property distributions depending on the search mode, separately for TiO_2_ as well as Zr-IMAC coupled to Pho-Tip (Figure S7); identified sequences comparing full proteome and CIP-based phosphopeptide libraries without MBR in DIA-NN, separately for TiO_2_ as well as Zr-IMAC coupled to Pho-Tip (Figure S8) (Supporting Information.pdf) (PDF).
List of identified phosphopeptides in CIP-treated and mock samples, separately for human TiO_2_-enriched (phosphopeptides_human.xlsx) (XLSX) as well as yeast Zr-IMAC-enriched coupled to Pho-Tip (phosphopeptides_yeast.xlsx) (XLSX)
