# Supplementary material for: Pho-Tip: One-Pot Dephosphorylation for Rapid and Sensitive Analysis of DIA Phosphoproteomics Data
Source: Anal Chem. 2026 Feb 23;98(9):6867–73. doi: 10.1021/acs.analchem.5c07139 (PMC12980491; doi:10.1021/acs.analchem.5c07139)
Supplement: Supplementary file 1 [file ac5c07139_si_001.pdf]

Supporting Information

Pho-Tip: one-pot dephosphorylation for rapid and sensitive analysis of DIA phosphoproteomics data

Katharina D. Faisst[1,†], Kate Lau[1,†], Ludwig R. Sinn[1,2], Lukasz Szyrwiel[1] and Vadim Demichev[1,*]

[1]Quantitative Proteomics laboratory, Department of Biochemistry, Charité – Universitätsmedizin Berlin, 10117 Berlin, Germany

[2]Biochemistry and Systems Biology of the Metabolism laboratory, Department of Biochemistry, Charité – Universitätsmedizin Berlin, 10117 Berlin, Germany

[†]Equal contributions

[*]Correspondence: vadim.demichev@gmail.com

Table of Contents

Figure S1. CIP-treatment efficiently desphosphorylates peptides.

Figure S2. Analysis of "CIP-resistant" phosphopeptides exhibits less acidic contexts and a higher abundance.

Figure S3. Overlap of total number of detected peptide sequences.

Figure S4. Dephosphorylation improves the detectability of peptides.

Figure S5. Pho-Tip results in lossless dephosphorylation.

Figure S6. The effect of dephosphorylation on quantitative precision.

Figure S7. Precursor property distributions depending on the search mode.

Figure S8. Comparing full proteome and CIP-based phosphopeptide libraries without MBR in DIA-NN.

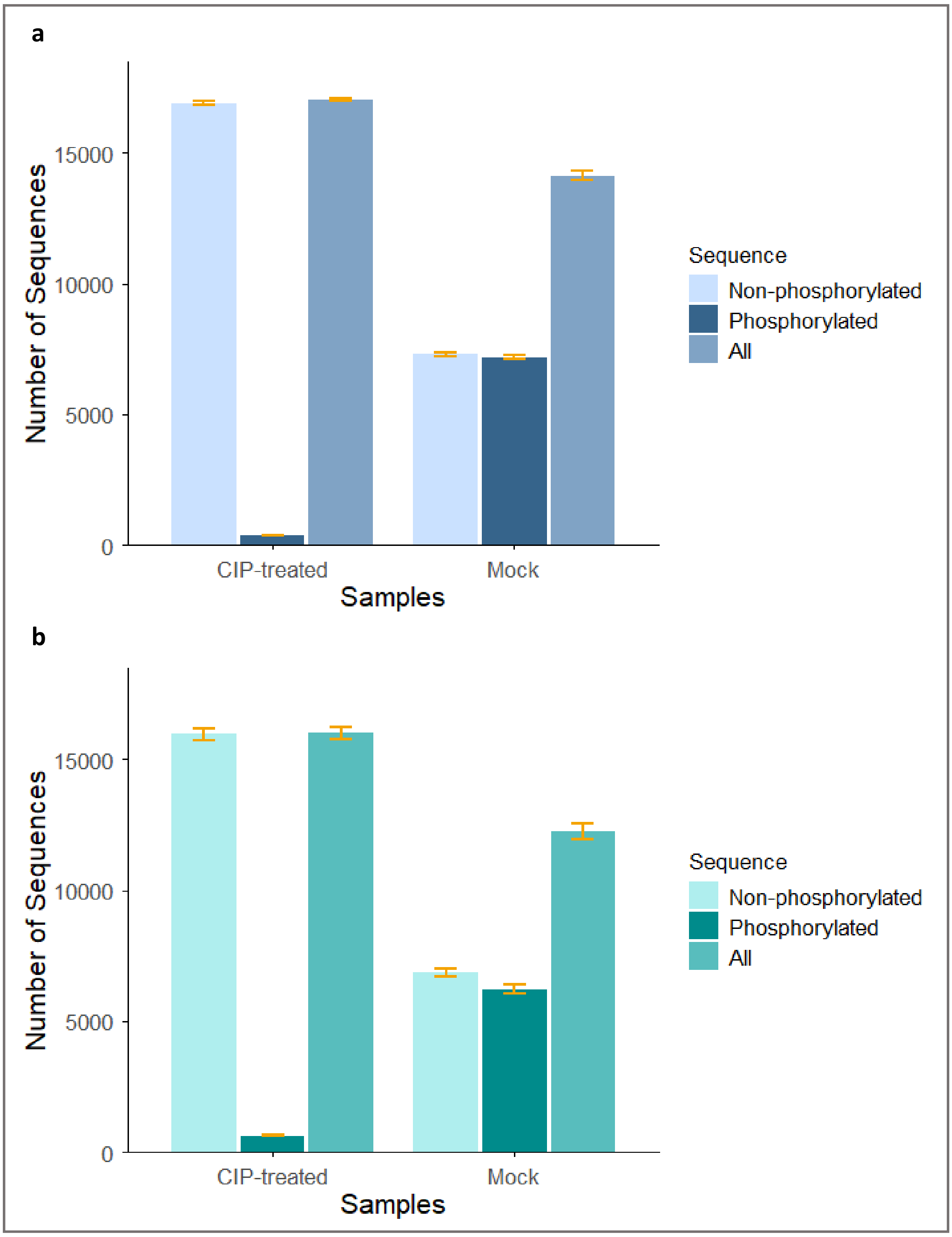

Figure S1. CIP-treatment efficiently desphosphorylates peptides. a) Number of phosphorylated, non-phosphorylated and all precursors in CIP-treated and mock samples for human TiO_2-enriched. Group means are shown, error bars indicate standard deviation. b) Number of phosphorylated, non-phosphorylated and all precursors in CIP-treated and mock samples for yeast Pho-Tip. Group means are shown, error bars indicate standard deviation.

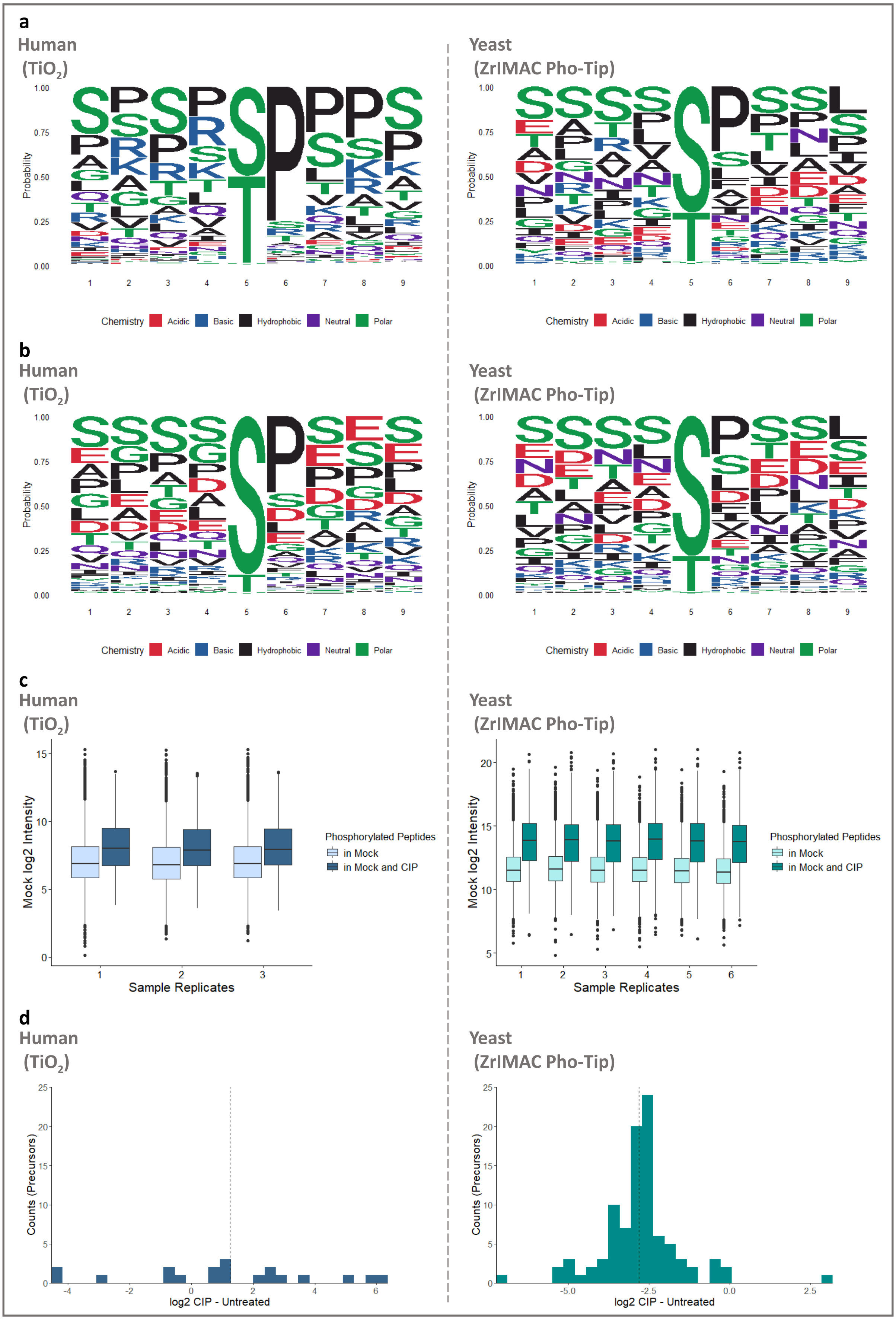
a
Human
(TiO2)
Yeast
(ZrIMAC Pho-Tip)
Probability
Chemistry Acidic Basic Hydrophobic Neutral Polar
b
Human
(TiO2)
Yeast
(ZrIMAC Pho-Tip)
Probability
Chemistry Acidic Basic Hydrophobic Neutral Polar
c
Human
(TiO2)
Mock log2 Intensity
Sample Replicates
Phosphorylated Peptides
in Mock
in Mock and CIP
Yeast
(ZrIMAC Pho-Tip)
Mock log2 Intensity
Sample Replicates
Phosphorylated Peptides
in Mock
in Mock and CIP
d
Human
(TiO2)
Counts (Precursors)
log2 CIP - Untreated
Yeast
(ZrIMAC Pho-Tip)
Counts (Precursors)
log2 CIP - Untreated

Figure S2. Analysis of "CIP-resistant" phosphopeptides exhibits less acidic contexts and a higher abundance. a) Motif analysis of still phosphorylated peptides detected in CIP-treated samples for human TiO_2-enriched and yeast Pho-Tip. b) Motif analysis of all phosphorylated peptides detected in mock samples for human TiO_2-enriched and yeast Pho-Tip. c) Normalised intensities of sequences of phosphopeptides detected in CIP-treated and mock samples compared to those that are dephosphorylated after CIP treatment, for human TiO_2-enriched and yeast Pho-Tip. d) Distribution of log2-transformed differences in integrated MS1 precursor intensities of CIP-resistant phosphopeptides in CIP-treated samples compared to the same phosphopeptides in mock samples for human TiO_2-enriched and yeast Pho-Tip, filtered for PEP $\leq$ 1%, Ms1.Profile.Corr $\geq$ 90%. The median values (1.24 human and -2.82 yeast) are indicated with dotted lines.

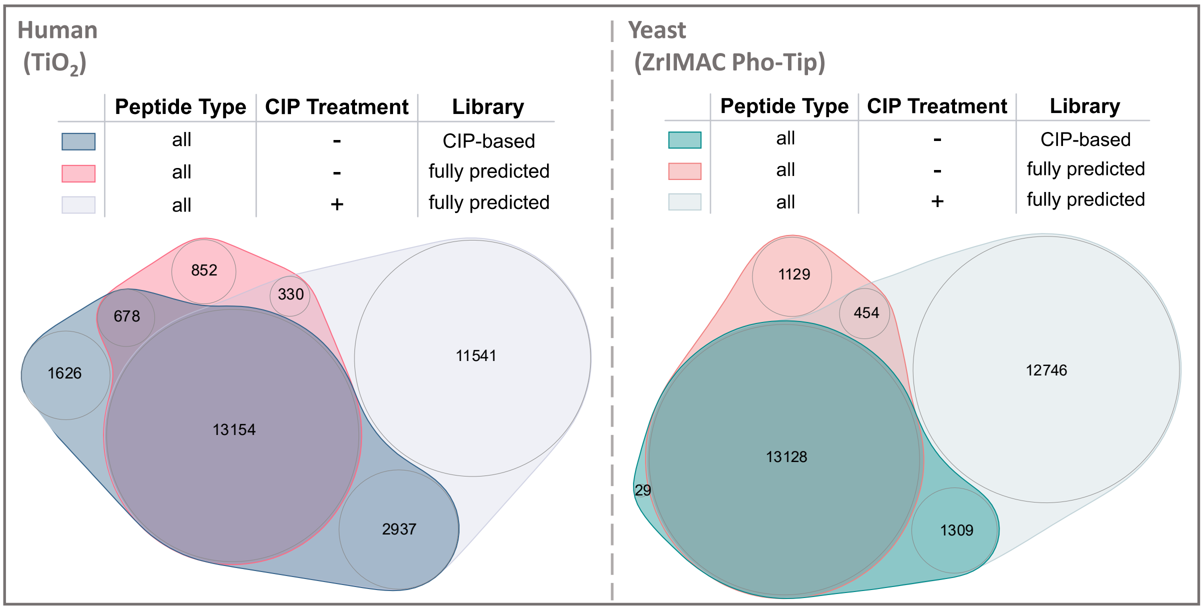

Figure S3. Overlap of total number of detected peptide sequences. Overlap of peptide sequences identified in mock samples, analysed with either fully predicted (red) or CIP-based library (blue/green), and peptide sequences in CIP-treated samples analysed with fully predicted library (grey), separately for TiO_2 as well as Zr-IMAC coupled to Pho-Tip.

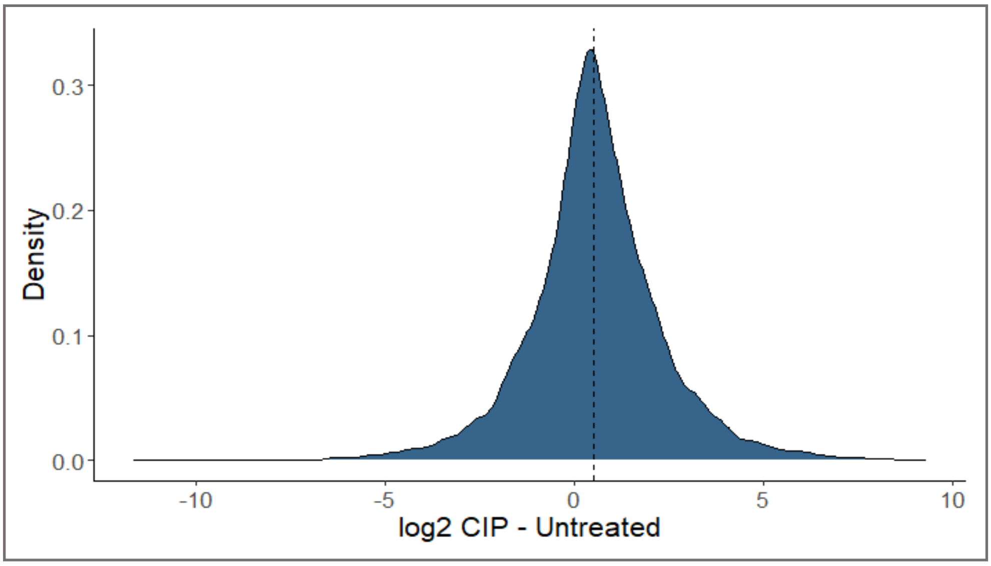

Figure S4. Dephosphorylation improves the detectability of peptides. Density distributions of log2-transformed integrated MS1 signal differences between unphosphorylated peptide sequences detected in CIP-treated samples and their phosphorylated counterparts in untreated samples for human TiO_2-enriched samples. Intensities of the latter were aggregated, for each stripped sequence, using the maximum value across matching precursors. Data were normalised to non-phosphorylatable peptides (those lacking Ser/Thr/Tyr residues) in the respective samples. The median value (0.31) is indicated with a dotted line.

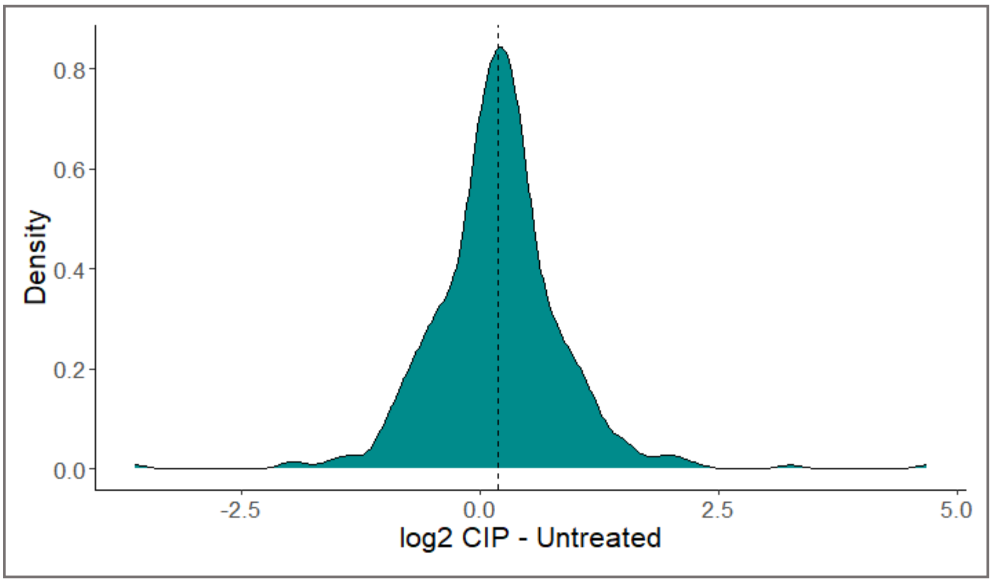

Figure S5. Pho-Tip results in lossless dephosphorylation. Density distributions of log2-transformed integrated MS1 signal differences between unphosphorylated peptide sequences detected in CIP-treated Pho-Tip samples and their phosphorylated counterparts in untreated samples. Non-phosphorylatable peptides (those lacking Ser/Thr/Tyr residues) are shown. Intensities were aggregated using the maximum values. The median value (0.19) is indicated with a dotted line.

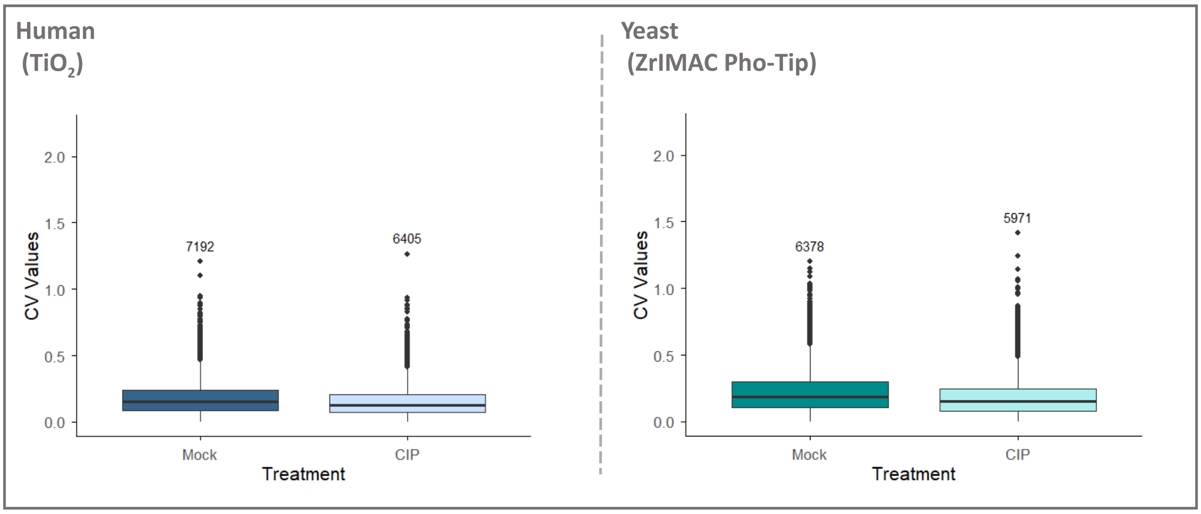

Figure S6. The effect of dephosphorylation on quantitative precision. Precursor-level CV values for jointly detected precursors by treatment, separately for TiO_2 as well as Zr-IMAC coupled to Pho-Tip. The boxes show the interquartile range (IQR) and the whiskers expand 1.5x the IQR.

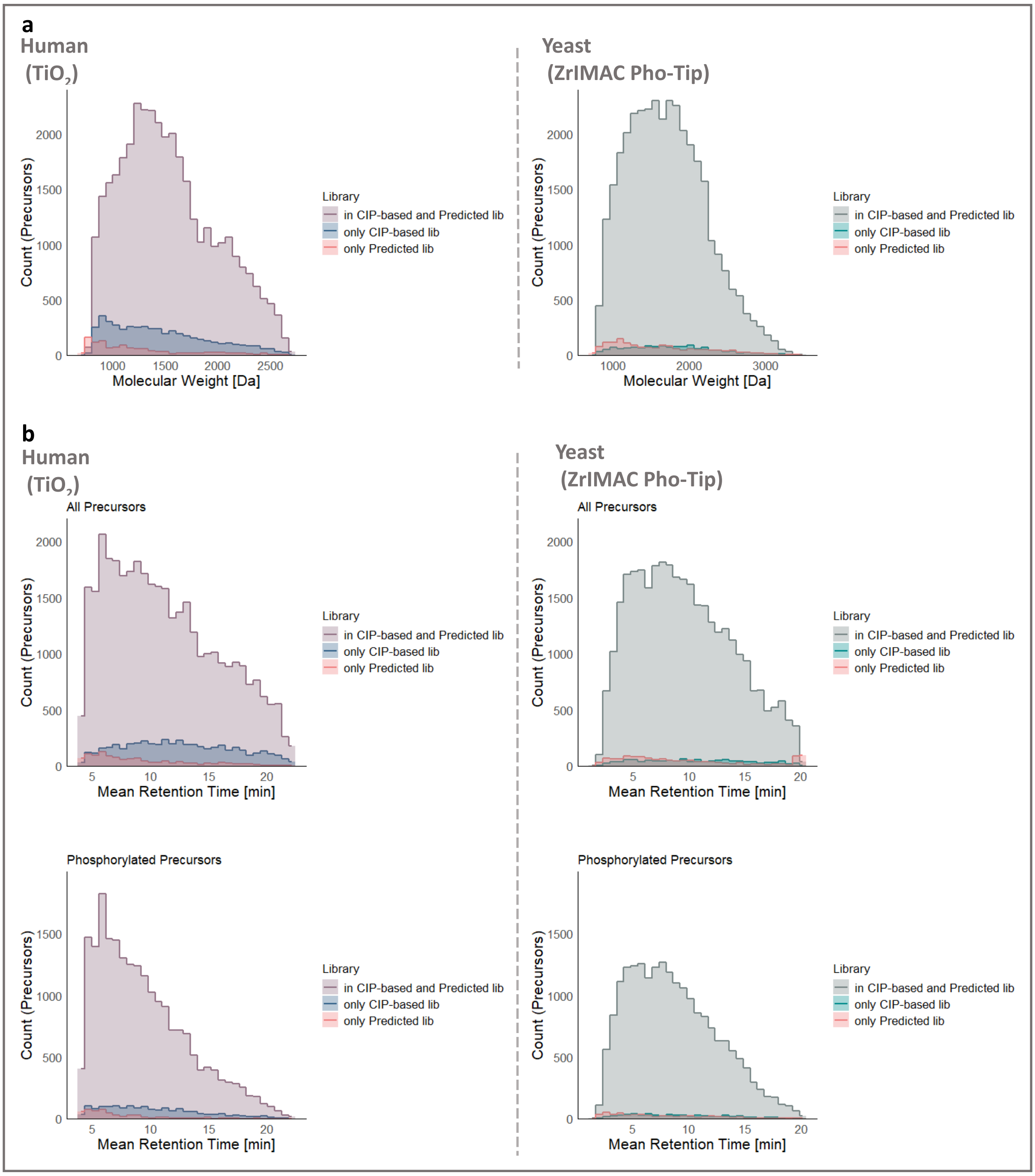

Figure S7. Precursor property distributions depending on the search mode. a) Distribution of molecular weight of precursors when searching with each library, separately for TiO_2 as well as Zr-IMAC coupled to Pho-Tip. b) Same for retention times.

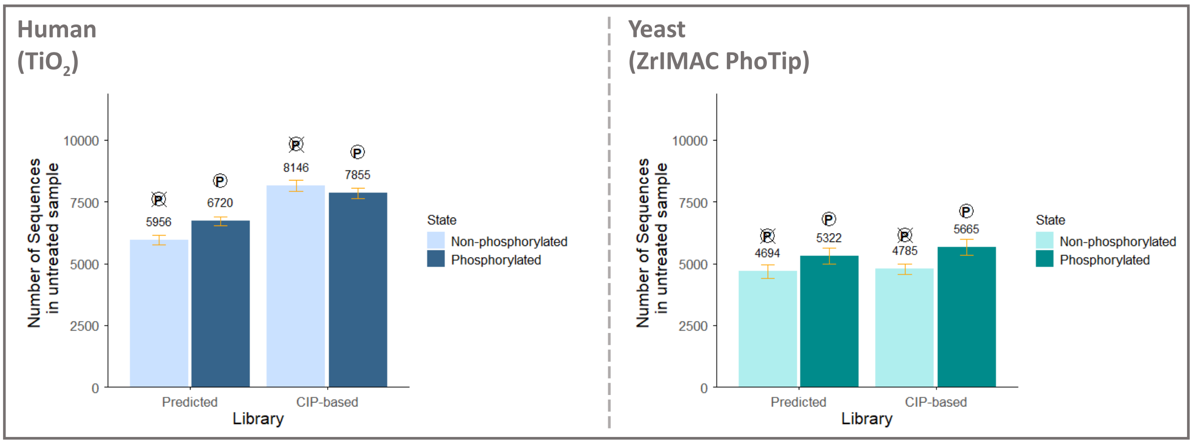

Figure S8. Comparing full proteome and CIP-based phosphopeptide libraries without MBR in DIA-NN. Number of identified sequences split by phosphorylation state (phosphorylated vs non-phosphorylated) is shown, separately for TiO_2 as well as Zr-IMAC coupled to Pho-Tip.
